# The Image of Group Fitness Instructors: An Intra- and Inter-Country Comparison Between Portugal and Romania

**DOI:** 10.3390/bs14121199

**Published:** 2024-12-13

**Authors:** Viorel Petru Ardelean, Vlad Adrian Geantă, Corina Dulceanu, Claudiu Bulzan, Sónia Brito-Costa, Guilherme Eustáquio Furtado, Ricardo Gomes, Fernando Martins, Francisco Campos

**Affiliations:** 1 Faculty of Physical Education and Sport, Aurel Vlaicu University of Arad, 2-4, Elena Dragoi, 310330 Arad, Romania; viorelpetruardelean@yahoo.com (V.P.A.); corina.dulceanu@yahoo.com (C.D.); claudiubulzan@yahoo.com (C.B.); 2 Physical Activities Research Center, 2-4, Elena Dragoi, 310330 Arad, Romania; 3 Doctoral School of Sport Science and Physical Education, Pitești University Center, National University of Science and Technology Politehnica Bucharest, 110253 Pitești, Romania; 4 Higher Education School, Polytechnic University of Coimbra, Rua da Misericórdia, Lagar dos Cortiços, S. Martinho do Bispo, 3045-093 Coimbra, Portugal; rimgomes@esec.pt (R.G.); fmlmartins@esec.pt (F.M.); francicampos@esec.pt (F.C.); 5 inED Centre for Research & Innovation in Education, Polytechnic University of Coimbra, Bencanta, 3045-601 Coimbra, Portugal; 6 SPRINT Sport Physical Activity and Health Research & Innovation Center, Polytechnic University of Coimbra, Bencanta, 3045-601 Coimbra, Portugal; 7 Research Centre for Natural Resources Environment and Society (CERNAS), Polytechnic Institute of Coimbra, Bencanta, 3045-601 Coimbra, Portugal; 8 Research Unit for Sport and Physical Activity (CIDAF), Faculty of Sport Sciences and Physical Education, University of Coimbra, 3040-256 Coimbra, Portugal; 9 Instituto de Telecomunicações, Delegação da Covilhã, 6201-001 Covilhã, Portugal

**Keywords:** fitness instructors’ image, customer satisfaction, cross-cultural comparison, fitness industry perception, fitness health

## Abstract

Fitness instructors play an essential role in fitness services, as they require both technical and interpersonal skills. A good image of a fitness instructor can be defined as having a pleasant appearance and good presentation in terms of hygiene and/or clothing used, which is appropriate for the context and characteristics of the participants. Their image significantly influences participants’ perceptions and satisfaction. The objective of this study was to conduct a comparative analysis between Romania and Portugal regarding the quality of services offered within group fitness classes, focusing on instructors. It involved 133 group fitness instructors and 210 participants from Romania and Portugal. An adaptation of the Szumilewicz questionnaire was used to assess the importance of the different attributes of the fitness instructor’s image. Statistical analyses included descriptive statistics, t-tests, and effect size to compare perceptions between countries and groups. Romanian instructors and participants generally attributed more importance to the fitness instructor’s image compared to their Portuguese counterparts. Significant differences were found in attributes like physical fitness, technical execution, and communication. Instructors tended to overestimate the importance of their image compared to participants. The fitness instructor’s image is crucial in determining participant satisfaction and the perceptions of service quality. The innovative aspect of this study lies in its intercultural comparison, which highlights how cultural context influences the perception of a fitness instructor’s attributes, such as physical fitness, technical execution, and communication. The practical application of these findings lies in the suggestion that fitness professionals should tailor their approach, balancing technical skills and interpersonal communication to align with the cultural expectations of their participants. This culturally sensitive approach is essential for enhancing participant satisfaction and improving the overall quality of fitness services.

## 1. Introduction

Physical fitness is a fundamental indicator of our current and future health status [1]. In contemporary society, there is a growing emphasis on health and well-being, prompting individuals to adopt healthier lifestyles. A significant aspect of this trend is the pursuit of physical exercise under the guidance of a personal trainer or within group exercise programs led by fitness instructors [2]. In both cases, specialized fitness professionals are fundamental agents for optimizing individuals’ physical fitness [3].

Certification authorities agree that, in addition to a solid background in anatomy, physiology, or biomechanics, the essential skills of a fitness instructor should include social and relational skills [3]. Furthermore, fitness instructors may also benefit from understanding population lifestyles, having knowledge of chronic diseases, having the ability to develop exercise programs and perform program management, and providing nutritional advice, among other skills [4]. When specifically analyzing the technical intervention of fitness instructors in group exercise activities, the musical domain—understood as comprising a sense of rhythm, mastery of music and its counts, adherence to musical structure, and synchronizing exercise to music [5]—emerges as one of the core competencies required for this type of intervention.

The increasing popularity of fitness activities, along with the professional training and certification of instructors, has attracted attention from international specialists. In the fitness industry, the image of the instructor is of paramount importance due to its significant impact on participants’ perceptions [5,6], whether the participants are healthy or disabled individuals [7].

A study conducted by Glaveli et al. [8] on the quality of interaction of fitness instructors in building trust with participants suggests that the communication skills of fitness instructors are essential for developing client trust in fitness centers, with a positive impact on client satisfaction. In contrast, technical skills did not have a significant impact. Furthermore, this study identified client trust in the service provider as a key variable that partially mediates the relationship between instructors’ social skills and client satisfaction, underscoring the importance of maintaining a positive image.

The positive image of a fitness instructor can be a good predictor of the quality of the instructor. The tone of voice [9], instruction style [10], pedagogical feedback [11], or the social and relational skills they possess [8,12] are key aspects that are used by participants to distinguish between an unprepared instructor and a well-prepared one [5,13]. Also, how the instructor observes the participants or class can influence pedagogical intervention, which involves making decisions based on those observations and subsequent thought processes [14].

Dobrich and collaborators explored how fitness culture shapes instructors’ body norms and self-perceptions, emphasizing the impact on their performance and ability to motivate participants [15]. Their study critiqued restrictive body image standards in group fitness and recommended more inclusive practices to enhance satisfaction and effectiveness at both individual and collective levels. In this study, we define “image” as outlined by Campos and their collaborators [5] (p. 13), who describe it as “a professional with a pleasant appearance and good presentation, particularly in terms of hygiene and/or clothing, appropriate to the context and the characteristics of the participants”. From a complementary perspective, this study aims to gather data on additional characteristics beyond these, focusing specifically on personal/relational attributes (e.g., education, cordiality, empathy, and affinity with participants) and technical/pedagogical skills (e.g., proper technical execution and strong rhythmic and musical mastery).

Currently, gyms and fitness centers serve as fundamental pillars in the physical activity habits of people of all ages [16]. Over the past three decades, the fitness industry has experienced remarkable growth, evolving from small training facilities targeted at athletes to sports-oriented centers for the general population [17]. This industry has significant potential to attract members and contribute to the optimization of the population’s health [18]. Due to this expansion, there is a high demand for fitness activities and, consequently, a challenge in educating and training well-prepared fitness instructors.

Considering the development of the fitness industry in both Romania and Portugal, driven by an increasing population demand, it is essential to assess the quality of services provided by fitness centers. This evaluation encompasses the quality of materials, equipment, services, location, and quality of human resources, particularly of the fitness instructors. This study focuses on the image of group exercise fitness instructors (GEFIs) as a quality indicator, evaluated from both the perspective of the instructors themselves and that of the participants who use and pay for these services.

While the image of GEFIs has been explored in Portugal [3], this topic remains both novel and significant in Romania. As fitness activities continue to grow in popularity and diversify, fitness professionals face an increasing number of challenges. The significance of the GEFI image for professional success has also been examined in Poland [6] through a qualitative study that investigated various aspects of the GEFI profile, including general attributes (e.g., rhythm and musical skills) and specific image-related characteristics (e.g., a toned body and well-defined musculature).

Several studies in Romania focus on improving physical fitness and health through various methods, such as weight training [19], bodyweight exercises [20], and functional training [21]. Nationwide research in Romania emphasizes the significance of fitness activities in maintaining overall health and achieving optimal physical fitness. It identifies a range of group exercise activities (e.g., Zumba, Tae-Bo, Cycling, Batuka, Step-Aerobics, Aqua Fitness, Body Toning, Pilates, Tabata, HIIT, Kangoo Jumps, Jukari) that are increasingly attracting participants. The significance of professional training, both theoretical and practical—ideally through long-term programs such as a bachelor’s and master’s—is recognized as fundamental for success in the field.

Despite the ongoing growth of the fitness industry in Romania and Portugal, supported by robust scientific methodologies in training, our investigation offers a unique contribution to the existing literature in both countries. To date, no studies have specifically examined the self-perception of GEFIs or the customer perception regarding the image and behavior of GEFIs in gyms and fitness centers. This research aims to lay the foundation for future inquiries into these critical aspects. The significance of this study is further highlighted by its dual focus on intra-country analysis and inter-country comparison between Romania and Portugal.

The objectives of this study were (a) to characterize the importance attributed to the GEFIs in Romania, comparing perspectives between GEFIs and participants, and (b) to compare the perceived importance of the GEFI image among GEFIs and participants in both Romania and Portugal. We hypothesized that GEFIs in Romania would report lower levels of self-perception compared to their Portuguese counterparts and that participants in both countries would place a similar emphasis on the GEFI image, reflecting a cross-cultural understanding of GEFI behavior and its impact on customer satisfaction.

## 2. Materials and Methods

### 2.1. Study Design

This study can be classified as quantitative in nature with a quasi-experimental design, as it did not involve a control group, and the sample was not randomly selected [22]. A convenience sampling method was employed, involving GEFIs and participants from fitness centers in Portugal and Romania. Participants were selected based on their availability and willingness to participate, making this a non-probabilistic sampling approach. This method facilitated data collection from accessible populations in both countries.

### 2.2. Participants and Settings

A total of 133 GEFI and 210 fitness participants of group fitness activities participated in this study. The sample was divided as follows: 102 Portuguese GEFIs (45.10% female; age: 29.66 ± 5.95 years; experience: 5.26 ± 4.59 years) and 31 Romanian GEFIs (58.06% female; age: 32.10 ± 10.45 years; experience: 7.70 ± 7.91 years), as well as 150 Portuguese participants in group fitness activities (79.33% female; age: 37.72 ± 15.14 years; experience: 7.16 ± 7.66 years) and 60 Romanian participants (58.33% female; age: 37.36 ± 8.52 years; experience: 4.78 ± 3.79 years).

### 2.3. Operationalization

Before the study began, fitness centers were contacted to gain permission to approach GEFIs for participation. Subsequently, GEFIs were reached via phone and email to explain the study’s content, scope, and objectives, encouraging them to inform participants about its importance. To ensure data quality, several factors were standardized, including the characteristics of the data collection environment (e.g., a quiet, naturally lit, and comfortable setting) and the materials provided (e.g., high-quality printed questionnaires, availability of pens, and clear, concise instructions).

Participant conditions were managed to include informed consent, study clarification, and assurance of well-being. Collection dates were established, and participants were reminded on the study day about the theme, objectives, procedures, and questionnaire details. They were informed of the importance of honest responses and assured of anonymity and confidentiality. After addressing questions and obtaining consent, the questionnaire was administered at the end of the session, with both participants and GEFIs completing it.

### 2.4. Ethics Statement

This project, titled “Assessing Quality and Satisfaction in Health and Fitness Service”, received approval from the Research Ethics Committee under opinion No. D40/2024. This study adheres to the European General Data Protection Regulation (GDPR), ensuring compliance with data protection and participant privacy standards. In line with the Declaration of Helsinki and Exercise Science Guidelines, all measures were taken to ensure participant safety and well-being. The study posed no direct risks, and participant anonymity was maintained, respecting their cultural, ethical, social, moral, and religious values throughout the research.

### 2.5. Instruments

(a) Sociodemographic data were collected using a structured questionnaire specifically designed to capture key participant characteristics. The instrument included several sections: demographic information such as age, gender, and country of residence (Romania or Portugal), allowing for cross-country comparisons. Data were collected exclusively from urban centers. Educational background was assessed, with response options ranging from 9th and 12th grade to bachelor’s and master’s degrees. Additionally, work experience in the fitness industry was categorized into ranges: 1 to 3 years, 4 to 6 years, 7 to 10 years, and more than 10 years. Participants were also asked to report the number of gyms they had attended or worked at, classified as 1, 2 to 3, or 4 or more. The questionnaire was designed to be clear and easy to understand, with instructions provided for each section to ensure clarity.

(b) For data collection on the profile of the fitness image, the study employed the GEFI Scale developed by Szumilewicz et al. [8], which was translated, adapted, and validated for use in both Romanian and Portuguese, following the procedures outlined by Campos et al. [3]. The translation and adaptation process adhered to established guidelines in the specialized literature [23,24,25,26], ensuring the cultural and linguistic appropriateness of the instrument in both countries. Face validity was confirmed through expert review. The final version of the instrument comprised 15 items, measured on a 7-point Likert scale that assessed the perceived importance of each item, ranging from 1 (not important at all) to 7 (absolutely important). This scale provided a detailed measure of the importance attributed by respondents, yielding robust data for subsequent analysis.

### 2.6. Analysis

The importance attributed to the GEFI image by participants and GEFIs was assessed using descriptive statistics (M ± SD). To facilitate group comparisons, independent samples t-tests were conducted, adhering to the assumptions of normality and homogeneity [27]. The normality of each univariate dependent variable was evaluated using the Kolmogorov–Smirnov normality test. In instances where normality was not confirmed, we invoked the Central Limit Theorem, allowing us to assume normality due to the sufficiently large sample size [27,28]. Levene’s test was utilized to verify the assumption of the homogeneity of variances [28].

For the comparison of means, the effect size (ES) was calculated using Cohen’s d index, with classifications as follows: very large (d > 1.0), large (0.5 < d ≤ 1.0), medium (0.2 < d ≤ 0.5), and small (d ≤ 0.2) [27]. In evaluating the reliability of the questionnaire, internal consistency was measured using Cronbach’s alpha coefficient [28], classified into the following categories: very good (α ≥ 0.9), good (0.8 ≤ α < 0.9), reasonable (0.7 ≤ α < 0.8), poor (0.6 ≤ α < 0.7), and unacceptable (α < 0.6). All statistical analyses were performed using IBM SPSS Statistics software (v.28, IBM, Armonk, NY, USA), with the significance level set at 5.00% (*p* < 0.05).

## 3. Results

Table 1 presents the demographic characteristics of the sample, including GEFIs and participants. Regarding age, most GEFIs were 34 years old or younger (Romania: n = 19, 61.29%; Portugal: n = 82, 80.39%). Among participants, 41.67% in Romania were aged 35–44 years (n = 25), while in Portugal, 33.33% were aged 45 years or older (n = 50).

In terms of education, the majority of GEFIs held at least a bachelor’s degree (Romania: n = 29, 93.55%; Portugal: n = 82, 80.39%). Among participants, 50.00% in Romania held a master’s or Ph.D. degree (n = 30), whereas in Portugal, 41.33% had completed only the 9th or 12th grade (n = 62).

Regarding professional or user experience, most GEFIs and participants reported having three years or less of experience [GEFIs: Romania: n = 14, 45.16%; Portugal: n = 43, 42.16%; participants: Romania: n = 26, 43.33%; Portugal: n = 58, 38.67%].

Finally, gym attendance patterns revealed that most GEFIs in both countries attended only one gym (Romania: n = 13, 41.93%; Portugal: n = 42, 41.18%). Among participants, the majority in Romania also attended only one gym (n = 33, 55.00%), while in Portugal, most attended two to three gyms (n = 64, 42.67%).

Table 2 compares the perceptions of Romanian GEFIs and participants regarding the importance of GEFI image attributes. The perceived importance of all items was high, with ratings ranging from 5.66 ± 1.40 (attractive dress and footwear) to 7.00 ± 0.001 (good hygiene) for both GEFIs and participants. Notably, GEFIs rated good physical fitness, education, cordiality, technical execution, and communication highly (all 7.00 ± 0.01), while participants rated only good technical execution as 7.00 ± 0.01.

Of the 15 items, only one—a sense of humor—was rated higher by participants compared to GEFIs, though no significant differences were found (*t* = −1.551; *p* = 0.128; *d* = 0.391; medium ES). Significant differences emerged for seven items: nice-sounding voice (*t* = 3.059; *p* = 0.003; *d* = 0.407; medium ES); well-toned body (*t* = 2.172; *p* = 0.033; *d* = 0.412; medium ES); good physical fitness (*t* = 4.370; *p* = 0.001; *d* = 0.690; large ES); education and cordiality (*t* = 2.491; *p* = 0.016; *d* = 0.379; medium ES); an original style (*t* = 3.187; *p* = 0.002; *d* = 0.604; large ES); clear communication (*t* = 3.013; *p* = 0.004; *d* = 0.496; medium ES); and a pleasant, attractive appearance (*t* = 2.223; *p* = 0.029; *d* = 0.497; medium ES).

Table 3 compares the Romanian and Portuguese contexts for both GEFIs and participants. For GEFIs, the importance attributed to the GEFI’s image was higher in Romania for 13 of the 15 items. Although there were no significant differences, two items showed higher importance in Portugal: good rhythmic and musical dominance (Portugal: 6.61 ± 0.62; Romania: 6.50 ± 0.81; *t* = 0.789; *p* = 0.431; *d* = 0.165; small ES) and dress according to the type of class being taught (Portugal: 6.19 ± 0.88; Romania: 6.10 ± 1.30; *t* = 0.371; *p* = 0.712; *d* = 0.091; small ES).

For GEFI, significant differences were found in 9 of the 15 items, with the following results: well-toned body with clearly defined musculature (*t* = −7.074; *p* = 0.001; *d* = 1.104; very large ES); good physical fitness (*t* = −10.164; *p* = 0.001; *d* = 1.135; very large ES); education and cordiality toward fitness participants (*t* = −3.968; *p* = 0.001; *d* = 0.454; medium ES); good hygiene (*t* = −5.438; *p* = 0.001; *d* = 0.615; large ES); good technical execution (*t* = −5.168; *p* = 0.001; *d* = 0.588; large ES); good energy and dynamism (*t* = −4.188; *p* = 0.001; *d* = 0.601; large ES); original style (*t* = −4.060; *p* = 0.001; *d* = 0.645; large ES); clear and objective communication (*t* = −6.486; *p* = 0.001; *d* = 0.738; large ES); and a pleasant, attractive, and good-looking appearance (*t* = −2.469; *p* = 0.015; *d* = 0.516; large ES).

For fitness participants, 14 out of the 15 items showed a higher importance attributed to the GEFI in Romania. Only one item, a nice-sounding voice, showed higher importance in Portugal, though the difference was not statistically significant (Portugal: 6.12 ± 1.04; Romania: 6.00 ± 1.19; *t* = 0.702; *p* = 0.484; *d* = 0.111; small ES).

Statistically significant differences were found in 13 out of 15 items for the fitness participants group, with large ES for the following items: well-toned body with clearly defined musculature (*t* = −6.403; *p* = 0.001; *d* = 0.850), good physical fitness (*t* = −6.291; *p* = 0.001; *d* = 0.691), good hygiene (*t* = −6.932; *p* = 0.001; *d* = 0.673), good technical execution (*t* = −7.208; *p* = 0.001; *d* = 0.693), good energy and dynamism (*t* = −4.119; *p* = 0.001; *d* = 0.523), clear and objective communication (*t* = −4.494; *p* = 0.001; *d* = 0.518), and a pleasant, attractive, and good-looking appearance (*t* = −3.996; *p* = 0.001; *d* = 0.536). Medium ES were observed for education and cordiality toward the fitness participants (*t* = −3.606; *p* = 0.001; *d* = 0.421), attractive dress and footwear (*t* = −3.028; *p* = 0.003; *d* = 0.463), sense of humor (*t* = −1.999; *p* = 0.047; *d* = 0.271), an original style (*t* = −2.763; *p* = 0.006; *d* = 0.416), good rhythmic and musical dominance (t = −3.991; *p* = 0.001; *d* = 0.494), and dress according to the type of class being taught (*t* = −2.574; *p* = 0.011; *d* = 0.339).

## 4. Discussion

This study aimed to investigate the significance attributed to GEFI in Romania while simultaneously comparing the perspectives of GEFI and fitness participants in Romania and Portugal. The primary objectives were twofold: (1) to evaluate how GEFI perceive their professional image in Romania in comparison to their Portuguese counterparts and (2) to examine whether fitness participants in both countries attribute a similar level of importance to the GEFI image, thereby providing insights into cross-cultural perceptions of GEFI behavior and its influence on customer satisfaction.

The sociodemographic analysis highlights key trends in the fitness sector across Romania and Portugal. Most GEFIs were younger, reflecting the youthful profile of fitness professionals [29], while Romanian participants were predominantly aged 35–44, indicating differences in fitness engagement. In both countries, the majority of GEFI held at least a bachelor’s degree, consistent with industry trends prioritizing academic qualifications [30]. Experience in the sector was limited, with most GEFI and participants reporting three years or less, reflecting high turnover rates and low retention among fitness users [29]. Additionally, Romanian participants predominantly attended a single gym (55%), whereas Portuguese participants more frequently attended two to three gyms (42.67%), suggesting more diversified fitness habits in Portugal, potentially explained by cultural differences [31].

In line with our hypotheses, the main results show that Romanian fitness GEFI reported lower levels of self-perception compared to their Portuguese counterparts, especially for key attributes such as “good physical fitness” and “clear communication”. Interestingly, Romanian GEFI rated these traits significantly higher, suggesting that they hold themselves to higher self-expectations.

Furthermore, while fitness participants in both Romania and Portugal acknowledged the importance of the GEFI image, significant differences emerged in the specific attributes they valued. These differences suggest that, although both groups place a strong emphasis on the GEFI image, cultural variations influence participants’ perceptions and expectations, ultimately affecting customer satisfaction in the fitness industry.

Building on previous findings by Soekmawati et al. [32,33] and Campos et al. [3], which highlighted the GEFI image as a critical factor in shaping participant experiences and expectations, our results reveal important nuances. While Szumilewicz et al. [6] suggested that the GEFI image holds only moderate importance for fitness participants when choosing specific group exercise activities—potentially indicating an overestimation by GEFIs—our study supports this perspective. Romanian GEFIs attributed significantly greater importance to 14 out of 15 GEFI image attributes compared to fitness participants. This notable discrepancy underscores a disconnect between GEFI’s self-perceptions and participants’ actual preferences, emphasizing the need for GEFIs to better align their understanding of their image with participant expectations to foster improved customer satisfaction.

A sense of humor appears to be underestimated by GEFIs when compared to participants’ perceptions. It is crucial for GEFIs to prioritize this trait in their professional interactions, as it fosters a more engaging and enjoyable atmosphere. Defined as being cheerful, fun, and smiling with a sense of humor [5] (p. 14), this attribute is strongly linked to participants’ satisfaction and overall experience.

Previous studies have emphasized the importance of interpersonal skills closely related to this trait, such as effective communication [34,35], empathy [36,37,38], sympathy [39,40,41], and maintaining a positive and approachable demeanor [5]. These qualities enhance the professional rapport between GEFI and participants, underscoring the need for greater focus on such soft skills in the practice of fitness instruction.

Significant differences emerged between Romanian GEFI and participants in their perceptions of the importance attributed to the GEFI image. Discrepancies were particularly evident in items such as a nice sounding voice; a well-toned body with clearly defined musculature; good physical fitness; education and cordiality toward participants; original style; clear and objective communication; and a pleasant, attractive, and good-looking appearance. In general, GEFIs rated these attributes as more important compared to participants. This aligns with previous findings by Szumilewicz et al. [6] and Campos et al. [3], suggesting that GEFIs may have an inflated perception of the role their image plays, further emphasizing the need for alignment with participant expectations.

When comparing Romania and Portugal, the importance attributed to the GEFI image is generally higher in Romania, with significant differences observed in nine attributes: a well-toned body with clearly defined musculature; good physical fitness; education and cordiality toward participants; good hygiene; good technical execution; good energy and dynamism; an original style; clear and objective communication; and a pleasant, attractive, and good-looking appearance. Among fitness participants, significant differences were identified in 13 attributes: a well-toned body with clearly defined musculature; good physical fitness; education and cordiality toward participants; good hygiene; good technical execution; attractive dress and footwear; a sense of humor; good energy and dynamism; an original style; good rhythmic and musical dominance; clear and objective communication; a pleasant, attractive, and good-looking appearance; and dressing appropriately for the type of class being taught. These findings suggest that the GEFI image is overestimated in Romania, both by GEFIs and participants, in comparison to Portugal [3].

In both Romania and Portugal, the image of the GEFI is a critical factor directly linked to the perceived quality of services offered in gyms and fitness centers. Significant differences were identified between the two countries in the perceptions of both GEFIs and participants, shaped by factors such as cultural and educational distinctions and the development of each country’s fitness market [31]. The findings of this study offer valuable insights for managing GEFI interventions, emphasizing the need to tailor promotion, marketing, communication, and training strategies to enhance participants’ perceptions and foster their active engagement in fitness activities.

A detailed understanding of these differences can support the development of a well-rounded and culturally sensitive GEFI image, strengthening the professional relationship with clients in Romanian gyms and fitness centers. Crucially, in both countries, GEFIs must adapt to the unique characteristics, needs, and preferences of participants, as highlighted in previous studies [3,5].

In recent years, gyms and fitness centers have embraced digitalization, incorporating innovations such as mobile applications [39,40,41], devices for monitoring performance and body metrics [42,43,44], and advanced functional training equipment like those using muscular electrostimulation [21], among others [45]. While these technological advancements undoubtedly add a new dimension to GEFI training and certification, we argue that the relational and pedagogical components of GEFIs remain paramount. These elements continue to be critical in shaping participants’ perceptions of service quality and satisfaction, ultimately driving loyalty and retention rates [5].

### 4.1. Strengths and Limitations

The findings of this study emphasize the significance of the image attributes of GEFIs in Romania and Portugal, highlighting key differences in perception between GEFIs and participants. This research creates opportunities for future cross-sectional or longitudinal investigations that can further explore these attributes and offer practical solutions for optimizing organizational strategies within the fitness industry.

However, this study has its limitations. One key limitation is the issue of sample representativeness; achieving full representativity was not possible in either country due to the convenience sampling method used. Additionally, there is a notable difference in the sample sizes between Romania and Portugal for both GEFIs and participants, which may have influenced the generalizability of the results.

Moreover, the cultural and contextual diversity between Romania and Portugal may have significantly impacted perceptions of the GEFI image. Local economic, social, and cultural factors, such as the stage of development of the fitness market and differing societal expectations regarding fitness professionals, likely shaped respondents’ views.

Another limitation is the potential subjectivity in participants’ responses. Individual and collective life experiences, personal preferences, and biases may have affected the evaluation of GEFI attributes, adding a layer of variability to the findings. These limitations underscore the importance of interpreting the results with caution and considering the contextual nuances that influence perceptions in different settings.

Finally, the lack of standardization in the GEFI intervention represents another limitation of this study. Data were collected from various fitness activities with distinct characteristics, leaving the choice of intervention strategies and methodologies entirely to the discretion of each GEFI. For instance, we did not control musical selection (e.g., genre, tempo, or cadence), despite evidence suggesting that music can significantly influence participants’ motivation, emotional response, and overall perceptions of the instructor [46]. Addressing this aspect in future research could provide deeper insights into the role of music in shaping participant experiences and perceptions of GEFI attributes.

### 4.2. Real-World Applications and Future Research Directions

Future studies should delve into specific factors influencing the differences identified in this research. These factors could encompass cultural, educational, and communicative aspects relevant to the fitness industry. For instance, a deeper analysis of how cultural and social values impact perceptions of the GEFI image could reveal best practices for GEFIs. Additionally, examining the role of the educational system in shaping GEFI professionals’ development in both countries could uncover discrepancies or similarities that affect study results.

Moreover, investigating the dynamics of communication between GEFIs and participants, including preferred interaction styles and communication channels, could enhance our understanding of each country’s fitness culture. A detailed exploration of these factors could lead to more comprehensive interpretations of the results and inform strategies for improving the fitness experience across diverse cultural contexts.

## 5. Conclusions

This study reveals that GEFIs and fitness participants in Romania and Portugal hold markedly different perspectives on the GEFI image. Specifically, Romanian GEFIs tend to overestimate the importance of their image attributes compared to participants, highlighting a disconnect that could negatively impact customer satisfaction. Through understanding these dynamics, GEFIs can better align their self-perceptions with participant expectations, thereby enhancing the overall workout experience. Addressing the limitations identified in this research and exploring future areas of study can help the fitness industry develop more effective strategies tailored to the diverse needs of participants, ultimately leading to improved customer satisfaction and retention.

## Figures and Tables

**Table 1:** Demographic characteristics of the sample: comparison between GEFIs and participants from Portugal (PT) and Romania (ROM).

		GEFI				Participants			
Variable	Category	Romania		Portugal		Romania		Portugal	
		n	%	n	%	n	%	n	%
Age (years)	Under 25	13	41.94%	20	19.61%	6	10.00%	36	24.00%
	25 to 34	6	19.35%	62	60.78%	12	20.00%	39	26.00%
	35 to 44	8	25.81%	16	15.69%	25	41.67%	25	16.67%
	45 years or older	4	12.90%	4	3.92%	17	28.33%	50	33.33%
Education	9th and 12th grade	2	6.45%	20	19.61%	5	8.33%	62	41.33%
	Bachelor’s degree	17	54.84%	53	51.96%	25	41.67%	55	36.67%
	Master’s or Ph.D.	12	38.71%	29	28.43%	30	50.00%	33	22.00%
Experience (years)	3 years or less	14	45.16%	43	42.16%	26	43.33%	58	38.67%
	4 to 6 years	5	16.13%	23	22.55%	22	36.67%	31	20.67%
	7 to 10 years	5	16.13%	19	18.63%	6	10.00%	29	19.33%
	More than 10 years	7	22.58%	17	16.66%	6	10.00%	32	21.33%
Gyms attended	1	13	41.93%	42	41.18%	33	55.00%	54	36.00%
	2 to 3	8	25.81%	39	38.23%	16	26.67%	64	42.67%
	4 or more	10	32.26%	21	20.59%	11	18.33%	32	21.33%

**Table 2:** Importance attributed to the GEFI image among Romanian study participants, focusing on differences between GEFIs and participants.

Item	GEFI	Participants	*t*	*p*	*d*
1. Nice-sounding voice.	6.58 ± 0.59	6.00 ± 1.19	3.059	0.003 **	0.407
2. Well tonified body, with clearly defined musculature.	6.27 ± 0.69	5.83 ± 1.21	2.172	0.033 *	0.412
3. Good physical fitness.	7.00 ± 0.01	6.74 ± 0.46	4.370	0.001 **	0.690
4. Education and cordiality toward the participants.	7.00 ± 0.01	6.91 ± 0.29	2.491	0.016 *	0.379
5. Good hygiene.	7.00 ± 0.001	7.00 ± 0.001	-	-	-
6. Good technical execution.	7.00 ± 0.01	7.00 ± 0.01	1.447	0.151	0.001
7. Attractive dress and footwear.	5.66 ± 1.40	5.13 ± 1.59	1.528	0.130	0.346
8. Empathy and affinity with the participants.	6.76 ± 0.43	6.63 ± 0.61	1.130	0.262	0.233
9. Sense of humor.	6.07 ± 1.02	6.39 ± 0.70	−1.551	0.128	0.391
10. Good energy and dynamic.	6.92 ± 0.25	6.84 ± 0.40	1.035	0.303	0.224
11. An original style.	6.60 ± 0.68	5.98 ± 1.16	3.187	0.002 **	0.604
12. Good rhythmic and musical dominance.	6.50 ± 0.81	6.41 ± 0.67	0.547	0.586	0.125
13. Clear and objective communication.	7.00 ± 0.01	6.87 ± 0.32	3.013	0.004 **	0.496
14. A pleasant, attractive and good-looking appearance.	6.50 ± 0.72	6.00 ± 1.12	2.223	0.029 *	0.497
15. Dress according to the type of class that they are teaching.	6.10 ± 1.30	6.02 ± 1.03	0.329	0.743	0.071

Statistically significant for * *p* < 0.05; ** *p* < 0.01.

**Table 3:** Comparison of the importance attributed to GEFI image, by GEFIs and participants, between Romania and Portugal.

	GEFI					Participants				
Item	Romania	Portugal	*t*	*p*	*d*	Romania	Portugal	*t*	*p*	*d*
1.	6.58 ± 0.59	6.34 ± 0.71	−1.630	0.106	0.350	6.00 ± 1.19	6.12 ± 1.04	0.702	0.484	0.111
2.	6.27 ± 0.69	5.03 ± 1.22	−7.074	0.001 **	1.104	5.83 ± 1.21	4.49 ± 1.70	−6.403	0.001 **	0.850
3.	7.00 ± 0.01	6.20 ± 0.80	−10.164	0.001 **	1.135	6.74 ± 0.46	5.99 ± 1.25	−6.291	0.001 **	0.691
4.	7.00 ± 0.01	6.80 ± 0.50	−3.968	0.001 **	0.454	6.91 ± 0.29	6.70 ± 0.56	−3.606	0.001 **	0.421
5.	7.00 ± 0.00	6.68 ± 0.59	−5.438	0.001 **	0.615	7.00 ± 0.00	6.59 ± 0.72	−6.932	0.001 **	0.673
6.	7.00 ± 0.01	6.72 ± 0.54	−5.168	0.001 **	0.588	7.00 ± 0.01	6.66 ± 0.58	−7.208	0.001 **	0.693
7.	5.66 ± 1.40	5.15 ± 1.41	−1.724	0.091	0.362	5.13 ± 1.59	4.31 ± 1.84	−3.028	0.003 **	0.463
8.	6.76 ± 0.43	6.62 ± 0.64	−1.108	0.270	0.234	6.63 ± 0.61	6.61 ± 0.67	−0.219	0.827	0.031
9.	6.07 ± 1.02	5.91 ± 0.65	−0.802	0.428	0.214	6.39 ± 0.70	6.15 ± 0.95	−1.999	0.047 *	0.271
10.	6.92 ± 0.25	6.62 ± 0.55	−4.188	0.001 **	0.601	6.84 ± 0.40	6.54 ± 0.63	−4.119	0.001 **	0.523
11.	6.60 ± 0.68	5.92 ± 1.14	−4.060	0.001 **	0.645	5.98 ± 1.16	5.48 ± 1.21	−2.763	0.006 **	0.418
12.	6.50 ± 0.81	6.61 ± 0.62	0.789	0.431	0.165	6.41 ± 0.67	5.89 ±1.17	−3.991	0.001 **	0.494
13.	7.00 ± 0.01	6.59 ± 0.63	−6.486	0.001 **	0.738	6.87 ± 0.32	6.58 ± 0.63	−4.494	0.001 **	0.518
14.	6.50 ± 0.72	6.08 ± 0.84	−2.469	0.015 *	0.516	6.00 ± 1.12	5.24 ± 1.52	−3.996	0.001 **	0.536
15.	6.10 ± 1.30	6.19 ± 0.88	0.371	0.712	0.091	6.02 ± 1.03	5.54 ± 1.54	−2.574	0.011 **	0.339

Statistically significant for * *p* < 0.05; ** *p* < 0.01.

## Data Availability

The original contributions presented in the study are included in the article; further inquiries can be directed to the corresponding author/s.
